# Characterization of Chromatin Remodeling Genes Involved in Thermal Tolerance of Biologically Invasive *Bemisia tabaci*

**DOI:** 10.3389/fphys.2022.865172

**Published:** 2022-05-20

**Authors:** Shun-Xia Ji, Xiao-Di Wang, Ze-Kai Lin, Fang-Hao Wan, Zhi-Chuang Lü, Wan-Xue Liu

**Affiliations:** ^1^ State Key Laboratory for Biology of Plant Diseases and Insect Pests, Institute of Plant Protection, Chinese Academy of Agricultural Sciences, Beijing, China; ^2^ Agricultural Genome Institute at Shenzhen, Chinese Academy of Agricultural Sciences, Shenzhen, China

**Keywords:** *Bemisia tabaci*, BRM, chromatin remodeling factors, ISWI, SWI/SNF, thermal stress

## Abstract

As an invasive species, *Bemisia tabaci* Mediterranean (MED) has notable potential to adapt to a wide range of environmental temperatures, which enables it to successfully spread after invasion and occupy habitats over a wide latitude range. It has been postulated that chromatin remodeling mechanisms are related to the rapid acquisition of adaptive traits and thermal resistance in invasive species; however, relevant experimental evidence is scarce. To identify the molecular characteristics and assess the role of chromatin remodelers in thermal stress within invasive MED and native Asia II 1 of the *B. tabaci* species complex, we identified 13 switching defective/sucrose non-fermenting (SWI/SNF) and 10 imitation switch (ISWI) family members in the *B. tabaci* genome, analyzed their molecular characteristics and structures, and identified key mutation sites between MED and Asia II 1, then cloned the catalytic subunits, and revealed the difference in thermal tolerance function. The results showed that the expression levels of *Bt-BRM-1* and *Bt-BRM-2* were significantly higher in MED than in Asia II 1 during heat stress, and *Bt-BRM-2* expression was significantly higher during cold stress. In addition, RNA interference results indicated that the two target genes had similar temperature tolerance function in the both two cryptic species. This study is the first to identify and analyze the molecular characteristics of SWI/SNF and ISWI family members and reveal their potential key roles in temperature tolerance in poikilothermic ectotherms. The results will assist in understanding the underlying temperature adaptation mechanism of invasive insects and will enrich stress adaptation research systems from an epigenetic perspective.

## Introduction

Epigenetics can induce phenotypic variation in numerous organisms via regulation of gene expression, by activating or silencing transposons, and by remodeling chromatin without altering the underlying nucleotide sequence (Bossdorf et al., 2008; Secco et al., 2015). Phenotypic variation caused by epigenetics is a reversible alteration that mediates the rapid and plastic responses of organisms to environmental perturbations, and it is considered to increase the capacity of organisms to adapt to environmental stresses (Johannes et al., 2009; Mirouze and Paszkowski 2011; Kumar 2018). Therefore, epigenetics has attracted unprecedented interest from researchers, particularly with respect to biological invasive species.

Chromatin remodeling is one of the most important components of epigenetics. In eukaryotes, packaging DNA into chromatin is essential for the organization and expression of the genome (Corona et al., 2007). However, the chromatin state determines the accessibility and transcriptional activity of specific genomic regions (Li et al., 2007). Intracellular evolution has produced a series of specific chromatin remodeling factors to ensure the dynamic binding of DNA and proteins in chromatin; these factors are multimeric proteins that utilize the energy generated from ATP hydrolysis to modulate the access of transcription factors and other regulatory proteins to genomic DNA by sliding nucleosomes and increasing chromatin fluidity (Corona and Tamkun 2004; Hopfner et al., 2012). Chromatin remodeling complexes are divided into four families according to the conserved domains of catalytic subunits: the switching defective/sucrose non-fermenting (SWI/SNF) family, the imitation switch (ISWI) family, the inositol requiring 80 (INO80) family, and the chromodomain helicase DNA-binding (CHD) family (Clapier and Cairns 2009).

The SWI/SNF family remodelers were initially purified from *Saccharomyces cerevisiae* and are composed of 8–15 subunits (Mohrmann and Verrijzer 2005). They have two forms in *Drosophila* (Clapier and Cairns 2009), namely brahma-associated protein (BAP) and polybromo-associated BAP (PBAP), both of which contain the same catalytic subunit, BRM. The catalytic enzyme BRM includes a helicase-SANT (HSA) domain and a C-terminal Bromo domain. The ISWI family of remodelers contain two to four subunits; they are characterized by ATPase ISWI as the catalytic core, and the catalytic enzyme ISWI is composed of an ATPase domain and a continuous HAND-SANT (SWI3, ADA2, N-CoR, TF III B)-SLIDE (SANT-like ISWI) domain (Clapier and Cairns 2009). The INO80 family of remodelers contain more than 10 subunits, and their ATPase contains a long insertion (Bao and Shen 2007a). Finally, the CHD family remodelers consist of one to ten subunits, and they were first purified from *Xenopus laevis* (Marfella and Imbalzano 2007).

Of these four families, SWI/SNF and ISWI remodeling factors have been widely studied for their biological responses to temperature stress. For instance, studies in *Drosophila* have shown that the nucleosome remodeling factor (NURF), which is a chromatin-remodeling complex that contains ISWI as its catalytic subunit, cooperates with the GAGA factor to mobilize nucleosomes on the promoter of heat shock genes, and facilitates the binding of heat shock transcription factors (HSFs) (Tsukiyama et al., 1994; Badenhorst et al., 2002). In *Arabidopsis*, BRM showed heat-induced transgenerational upregulation, and the binding of BRM to the HSFA2 region was enhanced by heat (Liu et al., 2019). Another study suggested that SWI/SNF remodelers and histone deacetylase interact and that both are involved in mediating the heat stress response of *Arabidopsis* (Buszewicz et al., 2016). Furthermore, the functional interplay between SWI/SNF and ISWI remodelers in the regulation of heat shock genes was determined in *Saccharomyces cerevisiae*, and inactivation of both remodelers could reduce the effective binding of HSF during the heat shock process (Erkina et al., 2010).

Insects are poikilothermic ectotherms, and subtle changes in ambient temperature can cause a series of physiological changes that affect their life-history parameters, such as their survival rate (Musolin 2007; Couret et al., 2014). Temperature is, therefore, one of the most important factors affecting the geographical distribution of ectotherms (Gutierrez and Ponti, 2014; Lancaster 2016). The thermal biology of insects is often investigated by assessing extreme thermal tolerance or by measuring the temperature performance of various traits across a permissive temperature range (Angilletta 2009; Sørensen et al., 2018). Using thermal tolerance or thermal performance as a proxy for thermal adaptation enables any association between a species and the thermal characteristics of their habitats to be detected (Andersen et al., 2015; MacLean et al., 2019).

The whitefly *Bemisia tabaci* (Gennadius) (Hemiptera: Aleyrodidae) is a notable pest in agriculture because it causes damage to more than 600 economic crops through phloem feeding, by transmitting plant viruses, and by depositing honeydew (Oliveira et al., 2001; Brown and Czosnek, 2002)*.* It is considered to be a species complex, as it comprises at least 36 morphologically indistinguishable species (De Barro et al., 2011; Liu et al., 2012; Wang and Yang 2017). The Mediterranean (MED) cryptic species was first detected in China in 2003, and it has successfully colonized and rapidly invaded most provinces. In contrast, the native species Asia II 1 is mainly distributed in the small natural areas of southern China (e.g., Hainan, Guangxi, and Zhejiang provinces), and it appears to have no tendency to spread to other areas (Chu et al., 2005; Hu et al., 2011; Liu and Liu 2012). As an invasive species, MED has the immense potential to adapt to a wide range of environmental temperatures, which allows it to successfully spread after invasion and to occupy habitats over a wide latitudinal range (Lee 2002; Cui et al., 2008; Yu et al., 2012).

Previous studies have shown that exposure of invasive whiteflies to thermal stress is accompanied by rapid alterations in their gene expression (Lü and Wan, 2008; Mahadav et al., 2009; Lü et al., 2014a; Dai et al., 2018a). Lü et al. (2014b) conducted heat shock experiments with MED and found that its survival rate was significantly improved within two generations. The authors speculated that this rapid increase in viability might be related to epigenetics. Another study (unpublished) analyzed transcriptome data and found that the expression levels of SWI/SNF and ISWI remodelers were significantly different between invasive and native whiteflies under temperature stress. Collectively, these results indicate that a possible explanation for the species’ rapidly increased temperature resistance is the regulatory plasticity of genes mediated by epigenetic mechanisms, such as chromatin remodeling. Although it has often been postulated that chromatin remodeling mechanisms are related to the rapid acquisition of adaptive traits and thermal resistance in invasive species, relevant experimental evidence is scarce.

In this paper, we report a series of experiments aimed at revealing the molecular characteristics and roles of remodelers in invasive MED and native Asia II 1. First, SWI/SNF and ISWI family members were identified in the *B. tabaci* genome, and their molecular characteristics, including the gene structures and conserved motifs, were analyzed using bioinformatics methods. Second, the catalytic subunits of the remodelers were cloned from MED and Asia II 1, and their tertiary structures and evolutionary relationships were analyzed. Third, the expression patterns of the catalytic subunits were examined after exposure to temperature stress in the two cryptic species. Finally, the function of the catalytic subunits in thermal stress was determined using the RNA interference (RNAi) method. These data reveal the novel function of chromatin remodeling factors in invasive *B. tabaci* MED and will help us to understand the temperature adaptation mechanism of invasive insects.

## Materials and Methods

### Insect Rearing

The invasive MED and native Asia II 1 used in this study were reared separately in the laboratory, and species were confirmed every month by the mitochondrial cytochrome oxidase I gene sequence according to the primer sequences reported by Ji et al. (2020). Whiteflies were reared on healthy cotton plants, *Gossypium hirsutum* (L.) (Zhong No. 287), in a greenhouse maintained at 26 ± 1°C at 50–60% relative humidity, and with a 14:10 h light: dark cycle.

### Identification and Characterization of SWI/SNF and ISWI Family Members in Whitefly Genome

Whole whitefly genome sequences were downloaded from the Whitefly Genome Project database (https://www.whiteflygenomics.org/cgi-bin/bta/index.cgi) (MEAM1, Chen et al., 2016). To identify the SWI/SNF and ISWI family members, blastp and tblastn were performed against the whitefly genome using the published remodelers from *Drosophila melanogaster* as queries with E-values ≤1e^−15^. All high-confidence sequences were submitted to the CDD database (https://www.ncbi.nlm.nih.gov/Structure/cdd/wrpsb.cgi), Pfam database (https://pfam.xfam.org/), and SMART (http://smart.embl.de/) to confirm the presence of conserved domains. Identified nucleotide and protein sequences of SWI/SNF and ISWI family members from the *B. tabaci* genome were submitted to EndMemo (http://www.endmemo.com/bio/gc.php), to calculate the GC content, and submitted to ExPASy (http://web.expasy.org/protparam/), to calculate the number of amino acids, molecular weights (MWs), and theoretical isoelectric points (PIs). The exon-intron structures of the remodelers were identified using TBtools (https://github.com/CJ-Chen/TBtools). The conserved motifs were predicted using the MEME program (https://meme-suite.org/meme/).

### Validation and Analysis of the Catalytic Subunit of SWI/SNF Complexes

Because catalytic subunit plays a critical role in the function of the SWI/SNF complexes, we validated and analyzed the gene sequences encoding the catalytic subunit in both MED and Asia II 1. Based on the catalytic subunit sequences obtained from the whitefly genome, specific primers for *BRM-1*and *BRM-2* were designed (listed in Supplementary Table S1) to validate the corresponding sequences in invasive MED and native Asia II 1 species. All polymerase chain reaction (PCR) amplifications were performed using FastPfu DNA Polymerase (TransGen, Beijing, China). The amplified fragments were purified using an AxyPrep DNA Gel Extraction Kit (Axygen, West Orange, NJ, United States), cloned into the pEASY-Blunt Vector (TransGen, Beijing, China), and subsequently sequenced.

The three-dimensional structure of the putative catalytic subunits was modelled using I-TASSER (https://zhanglab.ccmb.med.umich.edu/I-TASSER/). Conserved domains were predicted using SMART and CDD databases. Sequence alignments and similarity analyses were performed using DNAMAN version 7.0. Phylogenetic trees were constructed using the maximum likelihood method (based on the Whelan Goldman model) with 1000 bootstrap replications in MEGA 7.0 (Whelan and Goldman, 2001; Kumar et al., 2016). A color-coded pairwise identity matrix from amino acid sequences of remodelers belonging to different species was generated using SDT v1.2 software (Guo et al., 2021).

### Short-Term Temperature Stress Treatments

Pre-experimental results showed that the survival rate of whiteflies began to decrease significantly at temperatures below 0°C or above 40°C, i.e., *B. tabaci* could survive normally at 0–40°C. Therefore, temperatures of 0°C, 12°C, 35°C, and 40°C were used in the short-term temperature stress treatments to determine whether temperature stress affected the mRNA expression of the catalytic subunits (Dai et al., 2018b). As adult age is associated with different responses to thermal tolerance (Bowler and Terblanche, 2008), we standardized the adult age using only newly emerged whitefly adults that were younger than 3 h old. One hundred females and 100 males were placed together in a 1.5-mL centrifuge tube, and the tubes were placed in a constant-temperature environment (0°C, 12°C, 35°C, and 40°C) for 1, 3, and 5 h. Whiteflies maintained at 26°C were used as control groups. After conducting the temperature stress treatment, the whitefly adults were frozen immediately with liquid nitrogen and stored at -80°C. Each treatment was replicated thrice.

### RNA Isolation and Real-Time Quantitative PCR (qPCR)

Total RNA was extracted from whiteflies using TRIzol (Invitrogen, Carlsbad, CA, United States), and cDNA was synthesized using the cDNA Synthesis kit (TransGen, Beijing, China) following the manufacturer’s protocol. qPCR was performed using an ABI 7500 system (Applied Biosystems, Foster, CA). The amplification volume was 20 µL, comprising the following: 10.0 µL qPCR SYBR Green Master Mix (Yeasen, Shanghai, China), 0.8 µL primers (10 µM), 1.0 µL cDNA template, and 8.2 µL double distilled H_2_O. Three technical replicates were performed for each of the three biological replicates. The primers (including two reference genes, EF1-α and β-tubulin, to normalize the mRNA expression levels) are listed in Supplementary Table S1 (Miles 1965).

### RNAi

dsRNA used in this study was synthesized using the MEGAscript T7 High Yield Transcription Kit (Ambion, Austin, TX, United States) with specific primers (Supplementary Table S1). Feeding was performed using the parafilm clip nutrient solution method, and the dsRNA was diluted to 0.3–0.5 µg/µL in a 10% sucrose solution for feeding assay (Miles 1965; Lü and Wan, 2011). Moreover, we set control groups, including untreated whiteflies, which were fed with EGFP-specific dsRNA or 10% sucrose. After feeding for 3 h, partial samples were immediately frozen in liquid nitrogen and stored at -80°C for analysis of mRNA expression levels. The remaining whiteflies were collected immediately for conducting the temperature resistance experiments or temperature preference behavioral assays.

### Temperature Resistance After dsRNA Treatment

To analyze heat resistance, the knockdown times for adults exposed to high temperatures were measured (Bubli et al., 1998; Mitchell and Hoffmann 2010; Ma et al., 2014). Two whiteflies were randomly selected and placed in a 1.5-mL centrifuge tube, and the tube was maintained at a constant temperature of 45 ± 0.2°C in a water bath circulator (CC-106A, Huber Kältemaschinenbau GmbH). We measured the interval between the time the tube was placed in a water bath and the time when the whitefly lost control of its body and could not stand autonomously. For cold resistance, we measured the recovery time following a chill coma induced by cold shock (Gibert et al, 2001; Ma et al., 2014). Two newly emerged adults were randomly selected and placed in a 1.5-mL centrifuge tube, and the tube was exposed to -5 ± 0.2°C in a refrigeration bath circulator (K6-cc-NR, Huber Kältemaschinenbau GmbH) for 10 min; the recovery time was observed at 26 ± 0.2°C. Each treatment used 100 females and 100 males. Temperatures of 45°C and -5°C were selected based on pre-experiments that revealed them to be discrimination points for whitefly temperature tolerance (Ma et al., 2014).

### Statistical Analyses

Relative expression levels were calculated using the 2^**-**ΔΔCT^ method (Livak and Schmittgen 2001; Pfaffl 2001). Statistical analyses were conducted using the SAS 9.4 software package (SAS Institute, Cary, North Carolina), and a one-way analysis of variance followed by Duncan’s multiple range test was used to compare the differences between different treatments. Data are presented as mean ± standard error. Differences were considered statistically significant at *p*<0.05.

## Results

### Identification and Characterization of SWI/SNF and ISWI Family Members in Whitefly Genome

Remodelers are heteromeric proteins composed of several subunits(Clapier and Cairns 2009). In this study, 13 SWI/SNF family members were identified throughout the genome of *B. tabaci*, including three isoforms of subunit BRM; two isoforms of subunit BAP60 and BAP55; and one each of subunits OSA, BAP170, MOR, SNR1, BAP111, and Polybromo. For these subunits, the length ranged from 372 (Bt-SNR1) to 2079 amino acids (Bt-OSA), the MW was between 42.18 (Bt-SNR1) and 224.55 kDa (Bt-OSA), and the predicted PI ranged from 5.37 (Bt-moira) to 9.28 (Bt-BAP60-2). Moreover, 10 ISWI family members were identified from the whitefly genome, including four isoforms of subunit ISWI, as well as one each of subunits NURF301, ACF1, CHRAC16, CHRAC14, NURF55, and NURF38. For these subunits, the length ranged from 142 (Bt-CHRAC14) to 2691 amino acids (Bt-NURF301), the MW ranged from 15.45 (Bt-CHRAC14) to 300.48 kDa (Bt-NURF301), and the PI ranged from 4.61 (Bt-CHRAC14) to 9.32 (Bt-ISWI-3). Details of the SWI/SNF and ISWI remodelers obtained from the whitefly genome, including the gene length, GC%, and the predicted MW and PI, are presented in Table 1.

**TABLE 1:** Summary of SWI/SNF and ISWI family members found in whitefly genome.

Family	Subunit	Genome identifier	Strand	Length (bp)	ORF (bp)	GC%	Protein (AA)	MW (KDa)	PI
SWI/SNF	Bt-BRM-1	Bta07911	Minus	14938	4077	43.78	1358	154.68	6.52
	Bt-BRM-2	Bta01870	Plus	14421	4527	44.25	1508	171.85	6.30
	Bt-BRM-3	Bta14202	Minus	24103	4362	44.02	1453	166.68	6.39
	Bt-OSA	Bta14169	Minus	105077	6240	49.82	2079	224.55	6.62
	Bt-BAP170	Bta03801	Minus	42457	5238	50.02	1745	187.97	7.03
	Bt-MOR	Bta13338	Minus	18547	2859	44.46	952	106.91	5.37
	Bt-BAP60-1	Bta09866	Minus	51757	1377	43.50	458	52.71	9.18
	Bt-BAP60-2	Bta12826	Plus	13418	1473	44.87	490	56.60	9.28
	Bt-SNR1	Bta09680	Plus	11626	1119	44.95	372	42.18	5.38
	Bt-BAP111	Bta05888	Minus	37454	3693	54.83	1230	126.41	6.90
	Bt-BAP55-1	Bta05147	Minus	1310	1310	42.94	436	48.53	5.49
	Bt-BAP55-2	Bta08465	Plus	1310	1310	42.26	436	48.54	5.49
	Bt-Polybromo	Bta01538	Minus	145009	5028	42.34	1675	191.88	6.02
ISWI	Bt-ISWI-1	Bta10280	Minus	3068	3069	42.23	1022	118.86	8.36
	Bt-ISWI-2	Bta01732	Minus	5962	3708	41.02	1235	142.20	6.57
	Bt-ISWI-3	Bta08978	Minus	8511	3999	38.51	1332	154.23	9.32
	Bt-ISWI-4	Bta13898	Minus	2626	2448	39.46	815	94.47	8.70
	Bt-NURF301	Bta15867	Minus	135245	8076	42.50	2691	300.48	8.84
	Bt-ACF1	Bta10246	Plus	24566	4257	43.25	1418	162.90	6.27
	Bt-CHRAC16	Bta00621	Minus	4514	456	40.57	151	16.94	5.19
	Bt-CHRAC14	Bta01855	Minus	4314	429	40.79	142	15.45	4.61
	Bt-NURF55	Bta03516	Plus	10420	1284	42.83	427	48.19	4.72
	Bt-NURF38	Bta08309	Plus	14170	990	41.52	329	37.49	6.47

**FIGURE 1:**
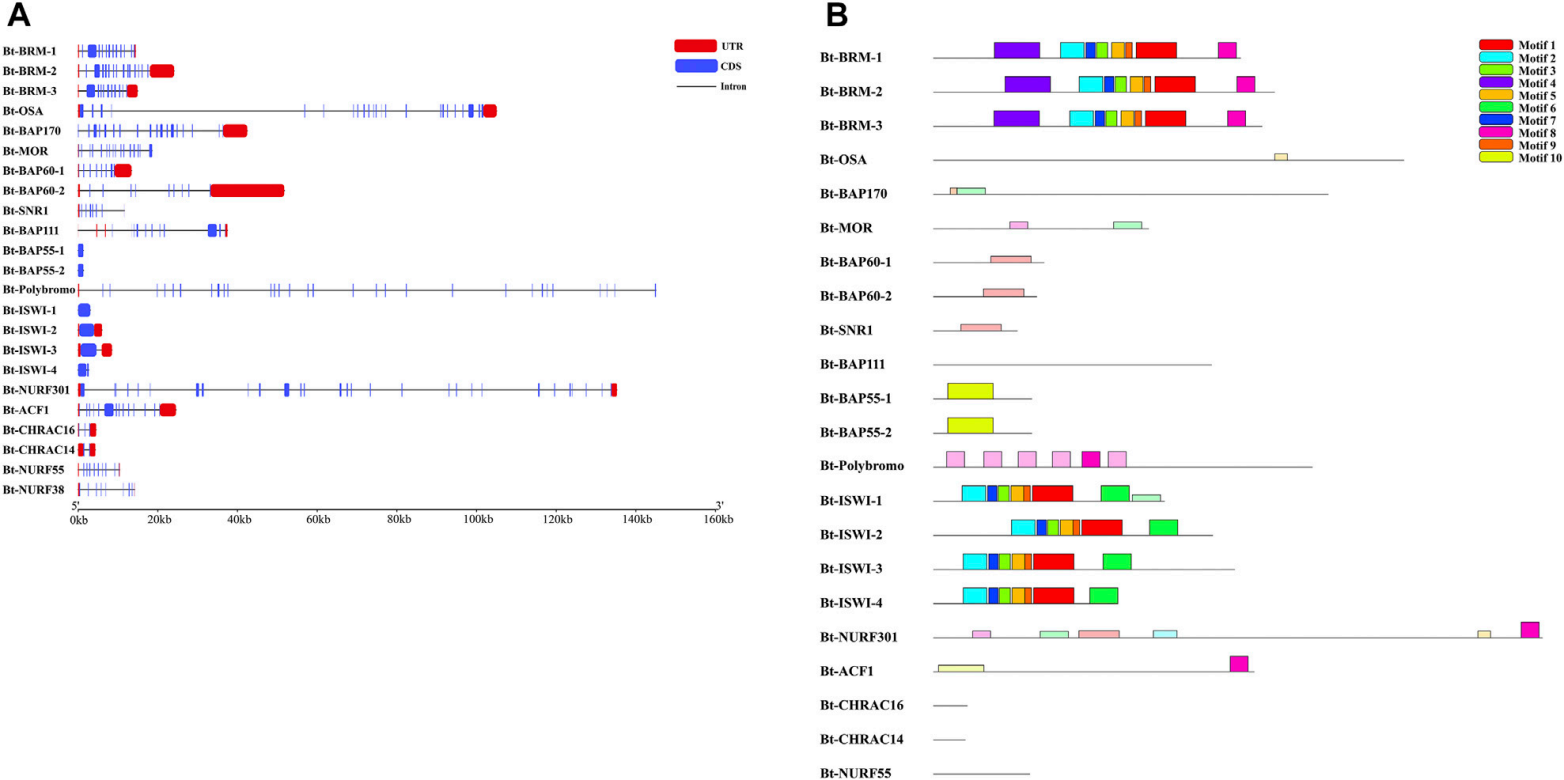
Gene structure and conserved motif analysis of remodelers in *Bemisia tabaci*. **(A)** Exon/intron organization of remodelers. Red boxes represent UTR, blue boxes represent exons and black lines represent introns. **(B)** Distribution of conserved motifs in remodelers. Ten putative motifs are indicated in different colored boxes. For details of motifs refer toTable 2.

The exon-intron structures of the SWI/SNF and ISWI family members were determined by comparing the full-length cDNA sequences with the corresponding genomic DNA sequences. The results showed that remodelers from the different families had completely different exon-intron structures and numbers (Figure 1A). The conserved motifs of chromatin remodeling factors were identified using the MEME program, and the lengths of these motifs varied from 41 (motif 7) to 200 amino acids (motifs 4 and 10) (Figure 1B). The details of the putative motifs are presented in Table 2. Moreover, based on analyses of the Pfam and CDD databases, we found that motifs 1, 2, 3, 5, and 7 collectively formed a highly conserved ATPase domain, and the sequences of motifs 4, 6, and 8 corresponded to the HSA, HAND, and Bromo domains, respectively.

**TABLE 2:** List of the putative motifs of SWI/SNF and ISWI remodelers.

Motif	Width	Best Possible Match
1	178	ZFLNMILHGLGJNLASADVVIIYDSDWNPQQDLQAQDRAHRIGQKKZVRVFRLITE
		NTVEEKIJERAEVKLRLDKLVIQQGRLDQKKTGLEKQLIRNCGKMVJLDKJLPKLK
		EQDSRVLIFCQMTRMMDILEDYFNWRGYKYCRLDGSTPHEDRGELJKKFNAPDSD
		KFLFLLSTRAG
2	104	CPSLKAVALKGDQEVRRAFIVDESPSYIKNGELRDYQIRGLNWLISLYENGJNGILAD
		EMGLGKTJQTISLJGYLKEYRKINGPHLIIVPKSTLSNWVNEFEKW
3	49	EFESPHRLLLTGTPLQNNLHELWALLNFLLPDVFKSASDFDAWFNANFC
4	200	RLAFLLSQTDEYISNLTEMVKQHKVEQKRKAERKRRQKHQEYLAAILQHCKDFKE
		YHRNNQARIVRLNKAILSYHVNAEREQKKEQERIEKERMRRLMAEDEEGYRKLID
		QKKDKAHRMEELKNLPTTMSEDLRLKAQIELRALRVLNFQRQLRNEIIACTRRDST
		LETAVBMKAYKRVKRQGLKEARATEKLEKQQKJE
5	56	RLHAVLRPFLLRRLKSEVESQLPPKVEYKVYIDLSKMQREWYTKILSKDIDJTBGA
6	124	DJDKICKEVEDKTPDEVJEYSSVFWDRFQELRDCNRIQARLYELQGKEFRWWRKSV
		ELQAPLDPRLGDKAAKAQYEEQLKIDEAQPLTEDEJEEKEDLLTQGFTNWSKKDFR
		QFVKANEKYGRD
7	41	WNVCITSYEYVJREKSALKKFNWKYMIIDEAHRIKNEHSKL
8	79	RVLSEPFMKLPSRKELPDYYEVIKKPMDJKKILTKIDEGKYEGLDDLERDFMQSFKN
		AQLYNEEGSLIYEDSIVLQSVF
9	29	GKGEKMRLLNIJMQLRKCCNHPYLFDGAE
10	200	KEQVKEKEKPKWTRKRNLPEVTTSWHDYMVFEKYNVPAFFLVKNAVLAAFANGRA
		TALVVDSGATHTSAIPVHDGYVLSHSIVKSPLGGDYISMQCKQLFQGQDIEIIPSYLIGG
		TQNINTSSENTKYYVDTTVLHAARKGVEVQSYMKDGMIENWDLFEKVLDYTYSKCI
		QSDSEYHPVLMSESPWNTRTKREKLTELM

### Validation and Analysis of the Catalytic Subunit of SWI/SNF Complexes

Based on the sequences obtained from the whitefly genome, the catalytic subunits *BRM-1* and *BRM-2* were cloned from invasive MED and native Asia II 1. The open reading frame length and the deduced amino residue number of *Bt-BRM-1* cloned from the two cryptic species were consistent with the corresponding sequence identified in the whitefly genome. However, the *Bt-BRM-2* cloned from the two cryptic species had seven amino residues more than the corresponding sequence identified in the whitefly genome (206-212: PALNSTP). Moreover, various sites in the amino acid sequences were derived from *Bt-BRM-1* and *Bt-BRM-2* in MED and Asia II 1 (Table 3).

**TABLE 3:** Different amino acid sites of catalytic subunits between MED and Asia Ⅱ 1.

Amino Acid Site	MED	Asia Ⅱ 1	Similarity (%)
Bt-BRM-1 (53)	Glutamic acid (E)	Aspartic acid (D)	99.85
Bt-BRM-1 (137)	Glycine (G)	Valine (V)	
Bt-BRM-1 (203)	Isoleucine (I)	Methionine (M)	
Bt-BRM-1 (248)	Serine (S)	Proline (P)	
Bt-BRM-1 (487)	Threonine (T)	Alanine (A)	
Bt-BRM-1 (526)	Aspartic acid (D)	Asparagine (N)	
Bt-BRM-2 (57)	Serine (S)	Threonine (T)	99.41
Bt-BRM-2 (148)	Alanine (A)	Threonine (T)	
Bt-BRM-2 (155)	Alanine (A)	Valine (V)	
Bt-BRM-2 (179)	Proline (P)	Serine (S)	
Bt-BRM-2 (202)	Proline (P)	Leucine (L)	
Bt-BRM-2 (600)	Isoleucine (I)	Valine (V)	
Bt-BRM-2 (1194)	Aspartic acid (D)	Glutamic acid (E)	
Bt-BRM-2 (1408)	Leucine (L)	Alanine (A)	
Bt-BRM-2 (1476)	Alanine (A)	Threonine (T)	

**FIGURE 2:**
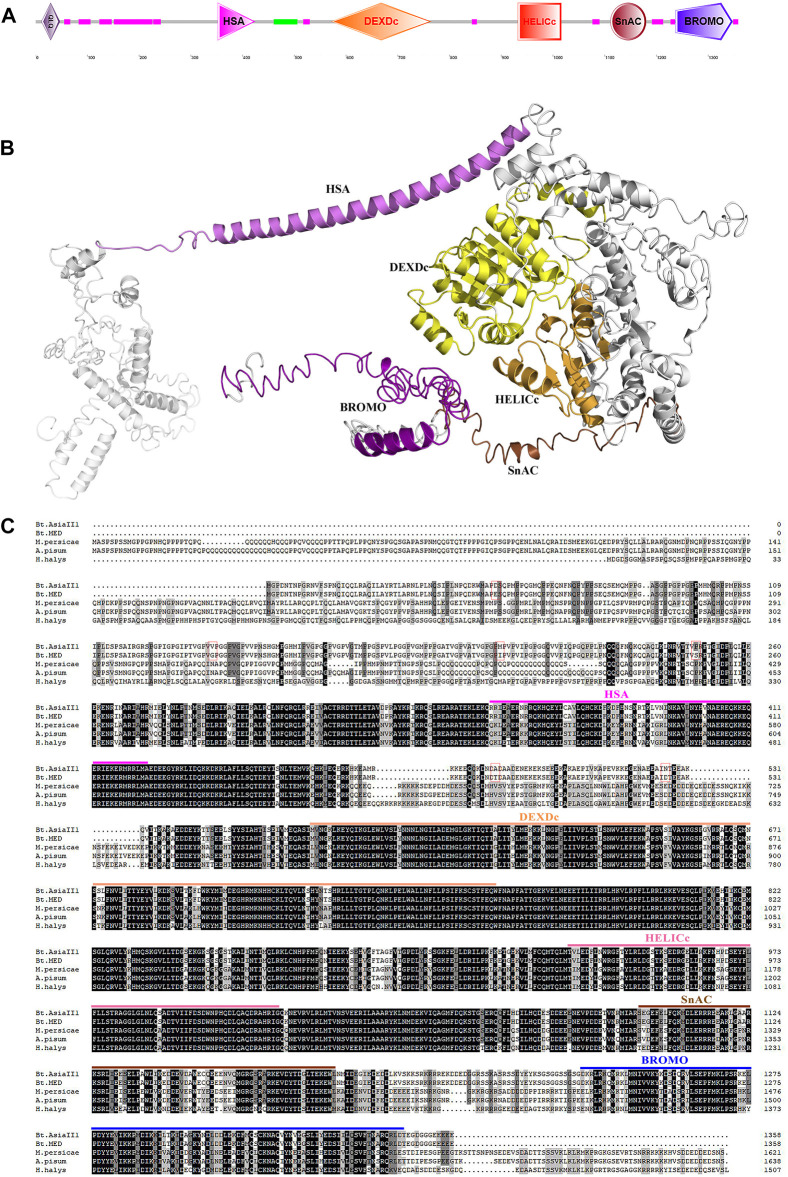
Conserved domains of Bt-BRM-1 in *Bemisia tabaci*. **(A)** Functional domains in Bt-BRM-1 protein. **(B)** The predicted tertiary structure of Bt-BRM-1. **(C)** Multiple alignments of the BRM protein from whiteflies and other insects. The conserved domains are highlighted in different colors and the amino acid sites that vary between MED and Asia II 1 are marked with red rectangles. Bt. Asia II 1: *Bemisia tabaci* Asia II 1; Bt. MED: *Bemisia tabaci* MED; M. persicae: *Myzus persicae* (XP_022166801.1); A. pisum: *Acyrthosiphon pisum* (XP_001947872.2); and H. halys: *Halyomorpha halys* (XP_014292008.1).

**FIGURE 3:**
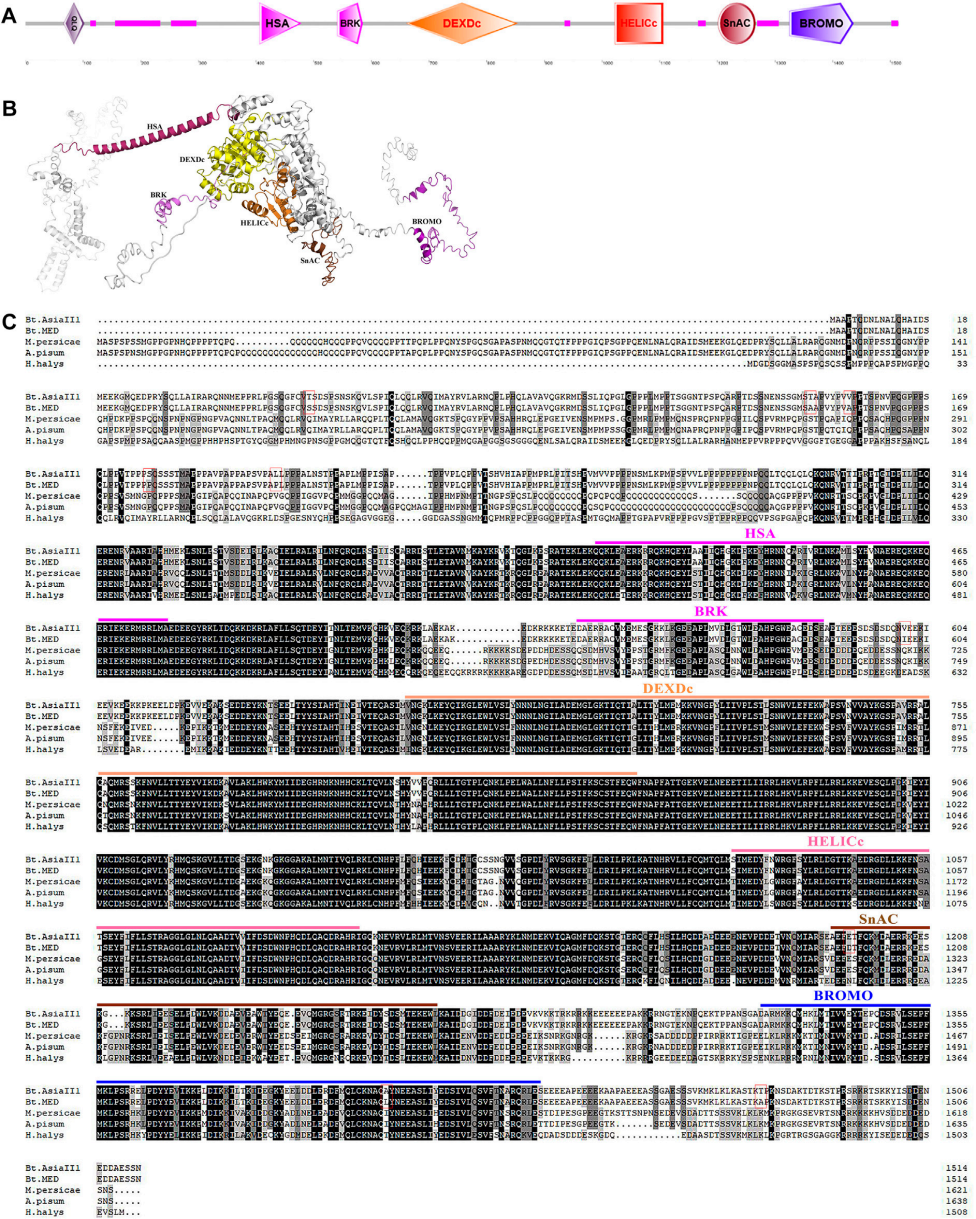
Conserved domains of Bt-BRM-2 in *Bemisia tabaci*. **(A)** Functional domains in Bt-BRM-2 protein. **(B)** The predicted tertiary structure of Bt-BRM-2. **(C)** Multiple alignments of the BRM protein from whiteflies and other insects. The conserved domains are highlighted in different colors and the amino acid sites that vary between MED and Asia II 1 are marked with red rectangles. Bt. Asia II 1: *Bemisia tabaci* Asia II 1; Bt. MED: Bemisia tabaci MED; M. persicae: *Myzus persicae* (XP_022166801.1); A. pisum: *Acyrthosiphon pisum* (XP_001947872.2); and H. halys: *Halyomorpha halys* (XP_014292008.1).

**FIGURE 4:**
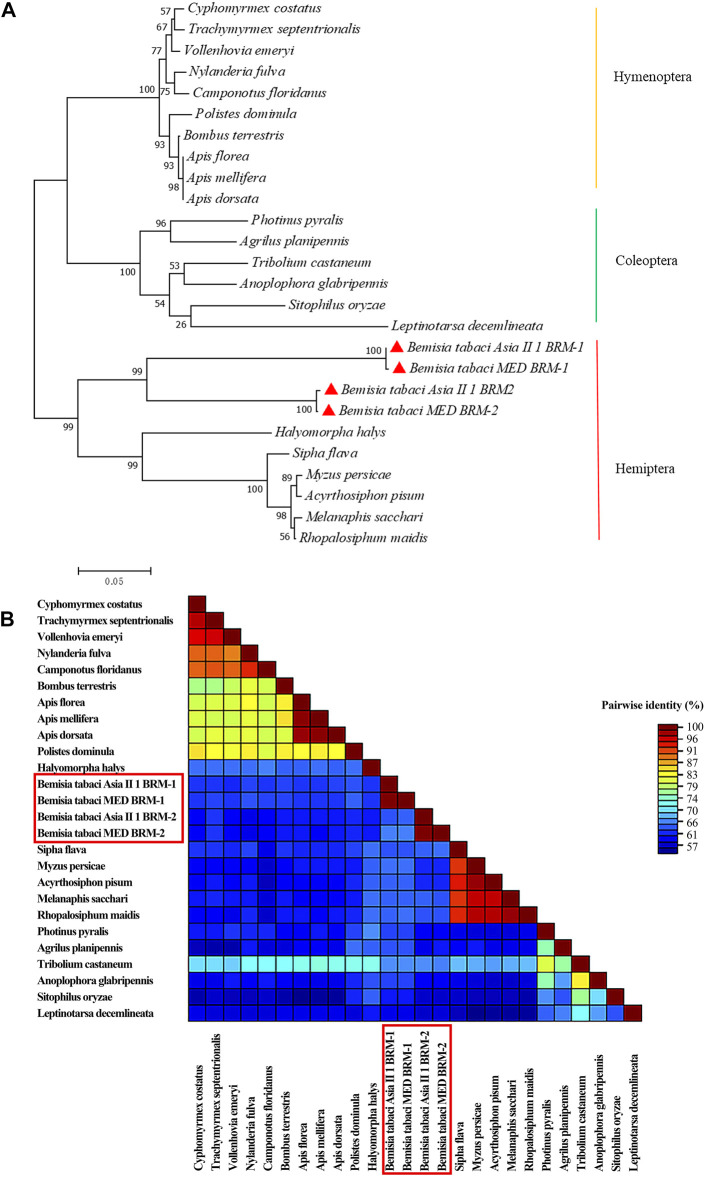
The relationships of Bt-BRM-1, Bt-BRM-2 and the homologous sequences from other species. **(A)** Phylogenetic analysis of the homologous sequences from various species performed using the maximum likelihood method based on the Whelan Goldman (WAG) model with 1,000 bootstrap replications. Bt-BRM-1 and Bt-BRM-2 are highlighted with red triangles, and the protein sequences which used for phylogenetic analysis are listed inSupplementary Table S2. **(B)** Color-coded pairwise identity matrix from amino acid sequences of remodelers from different species generated using SDT software. Bt-BRM-1 and Bt-BRM-2 are marked with red rectangles.

Conserved domain analysis indicated that both Bt-BRM-1 and Bt-BRM-2 had domain features unique to the SWI/SNF family: the HSA domain and the C-terminal bromodomain (Figures 2A, 3A). Moreover, I-TASSER was used to predict the three-dimensional structure of the proteins. The results showed that remodelers in *B. tabaci* had structures similar to those of yeast (Figures 2A, 3A)(Wagner et al., 2020). In addition, multiple sequence alignments of remodeler catalytic subunits from different species revealed that they were relatively conserved (Figures 2C, 3C). The similarity of Bt-BRM-1 and Bt-BRM-2 to other species exceeded 62.5 and 63.7%, respectively. Furthermore, the relationships between Bt-BRM-1 and Bt-BRM-2, and the homologous sequences from other species were analyzed (Figure 4), and remodelers of insects in the same order were clustered on the same branch of the phylogenetic tree.

### Expression Profiles of Catalytic Subunits Under Temperature Stress

**FIGURE 5:**
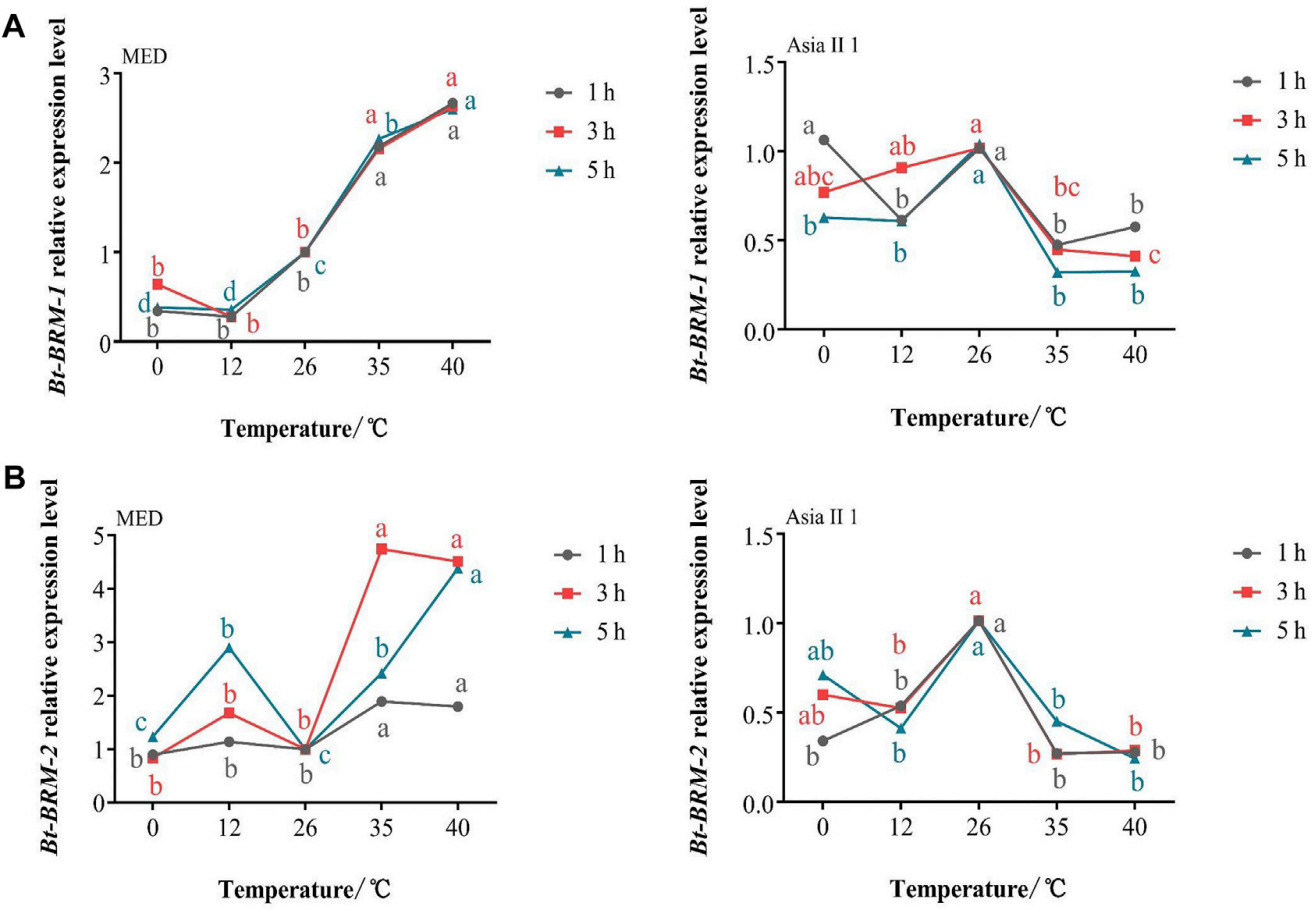
Variation trend of relative expression levels of *Bt-BRM-1* **(A)** and *Bt-BRM-2* **(B)** in MED and Asia II 1 after exposure to different temperatures. Different lowercase letters indicate significant difference at *p* < 0.05.

**FIGURE 6:**
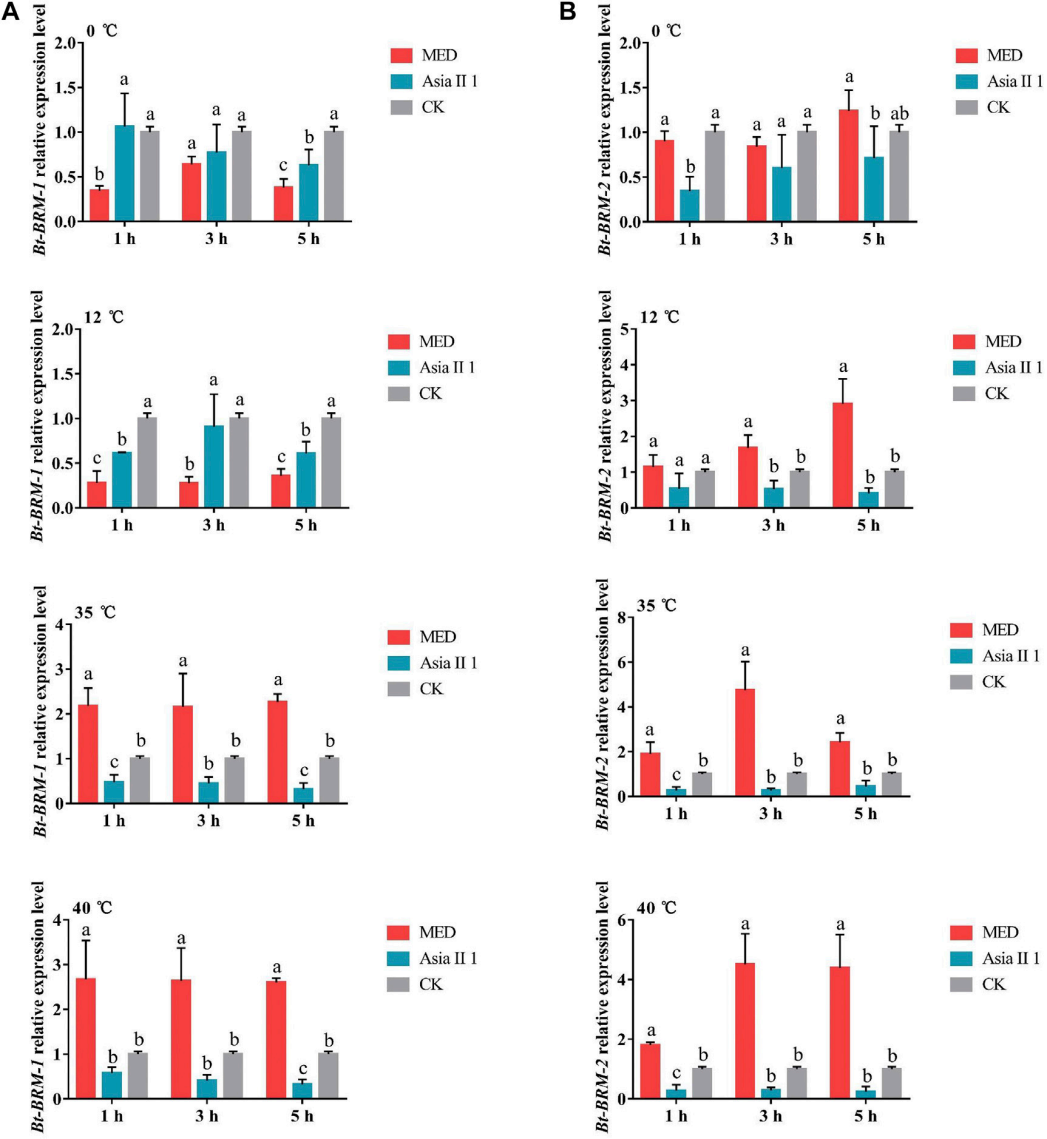
Relative expression levels of *Bt-BRM-1* **(A)** and *Bt-BRM-2* **(B)** in MED and Asia II 1 after exposure to different temperatures. Different lowercase letters indicate significant difference at *p* < 0.05.

To investigate whether temperature stress affects the mRNA expression of chromatin remodeling factors, whiteflies were exposed to hot and cold conditions for various time periods. Compared with individuals maintained at 26°C (the control temperature), the expression of *Bt-BRM-1* gene in MED and Asia II 1 was downregulated under cold shock conditions. The expression pattern was opposite in the two cryptic species (upregulated in MED, but downregulated in Asia II 1) under heat shock conditions (Figure 5A). The expression level of *Bt-BRM-2* in MED was significantly upregulated after exposure to short-term heat or cold conditions, whereas it was significantly downregulated in Asia II 1 (Figure 5B). Additionally, the mRNA levels of *Bt-BRM-1* and *Bt-BRM-2* in MED were significantly higher than those in Asia II 1 during heat stress, and the expression level of *Bt-BRM-2* in MED was significantly higher than that in Asia II 1 during cold stress (Figure 6). These data indicated that remodelers in whiteflies were strongly induced by heat or cold stress, and the expression profiles differed significantly between the invasive MED and native Asia II 1 species.

### Function of Chromatin Remodeling Factors During Thermal Stress

**FIGURE 7:**
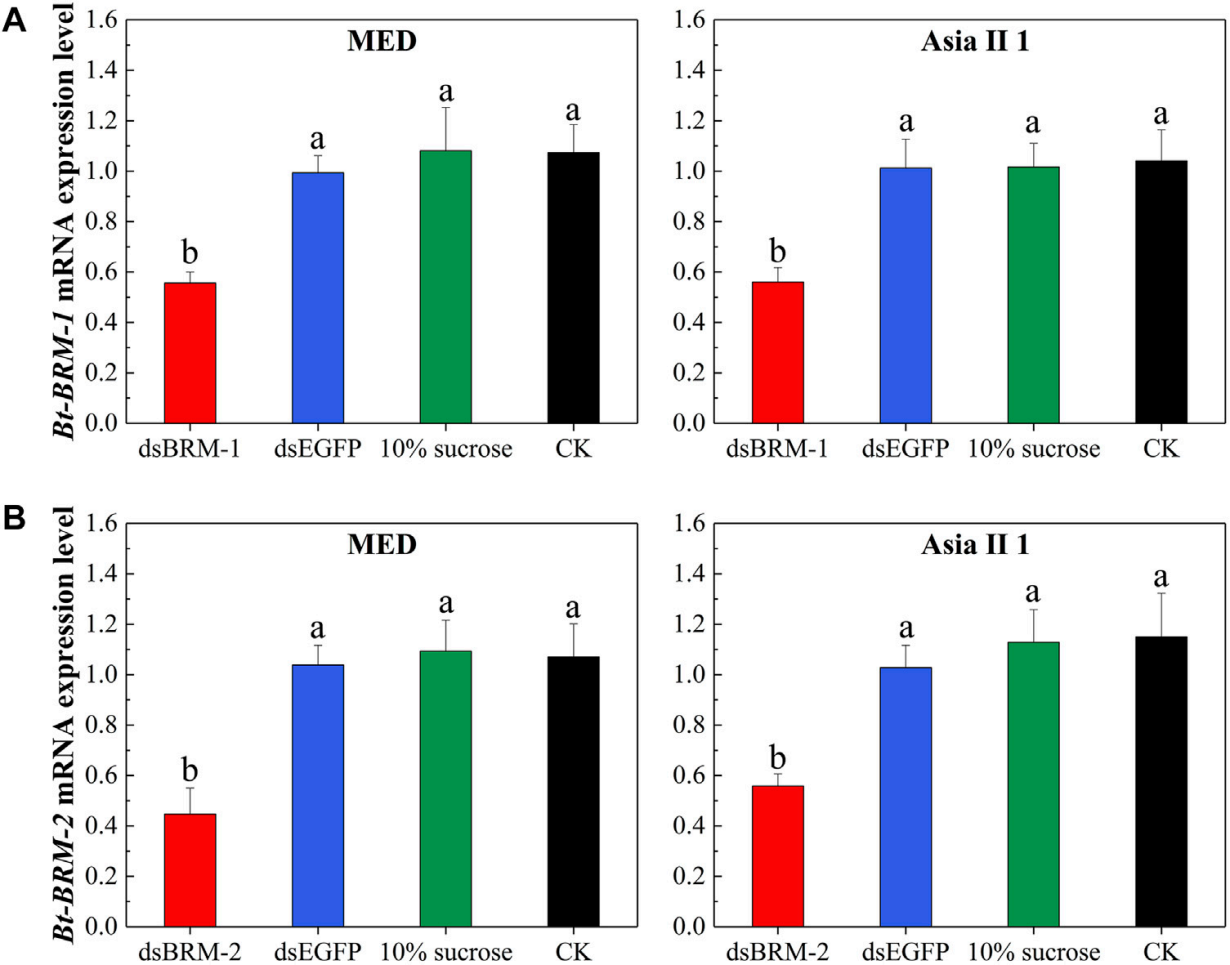
Effect of double-stranded RNA (dsRNA) treatment on expression levels of *Bt-BRM-1* **(A)** and *Bt-BRM-2* **(B)**. The expression levels of target genes in MED and Asia II 1 after feeding with dsRNA for 3 h were significantly decreased compared with the controls: whiteflies untreated (CK), fed *EGFP* dsRNA (dsEGFP), or given 10% sucrose. The expression levels are expressed as the mean ± SE. Bars with different lowercase letters are significantly different at *p* < 0.05.

To explore the function of catalytic subunits, we fed newly emerged whitefly adults with dsRNA to silence the target genes. The expression levels of target genes in MED and Asia II 1 after feeding with dsRNA for 3 h were significantly lower than those in the control groups (Figure 7). The primer sequences used to detect mRNA expression are listed in Supplementary Table S1.

**FIGURE 8:**
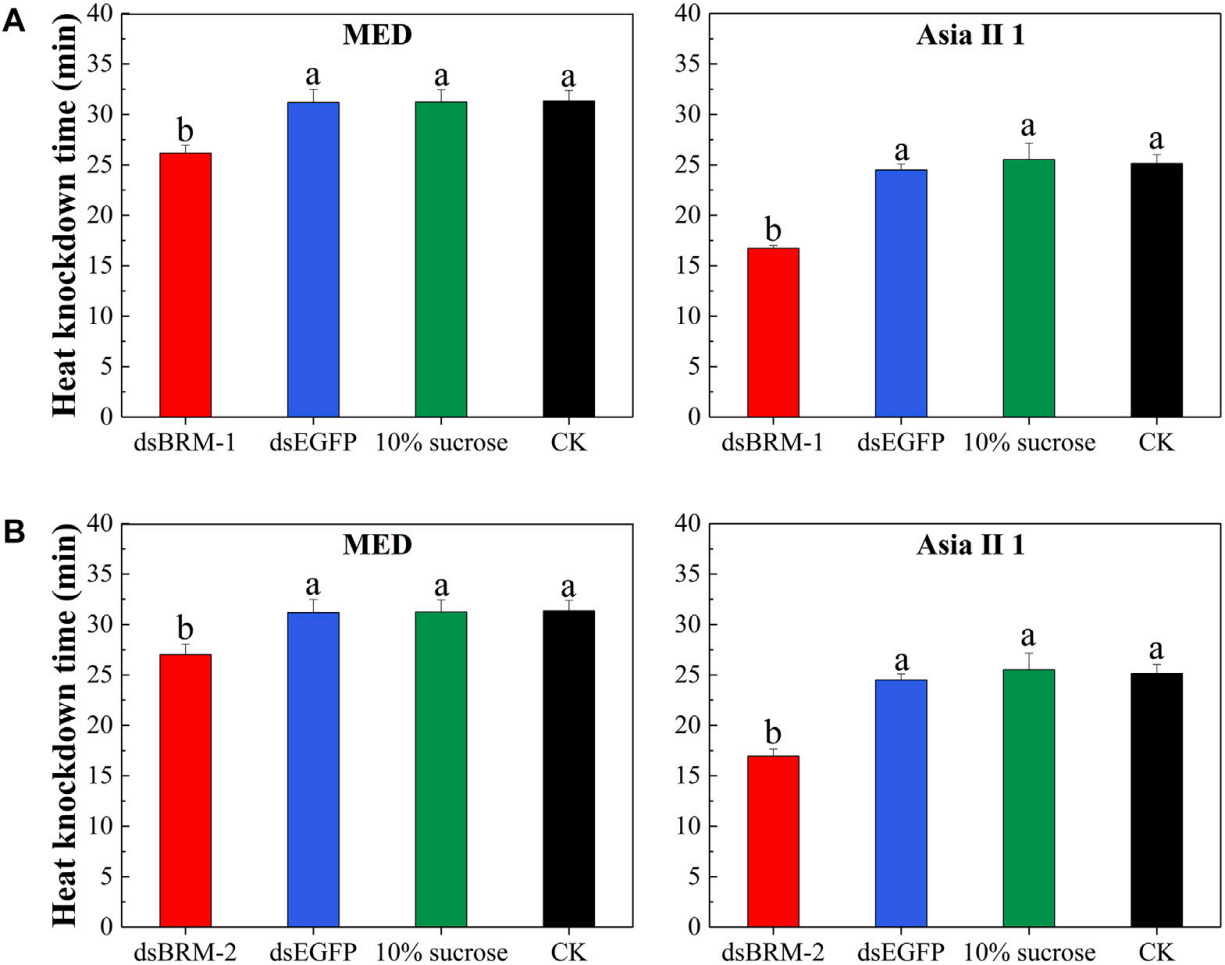
Mean heat knockdown time (TKD) after double-stranded RNA (dsRNA) feeding in whitefly adults. Compared with the control groups (whiteflies untreated (CK) or fed dsEGFP or 10% sucrose), TKD for MED and Asia II 1 after feeding with *Bt-BRM-1* **(A)** or *Bt-BRM-2* **(B)** dsRNA was significantly decreased. Moreover, heat resistance of the two cryptic species was reduced differently after interfering with the remodelers (details are listed inSupplementary Table S3). The data are presented as the mean ± SE; *n* = 200. Bars with different lowercase letters are significantly different at *p* < 0.05.

**FIGURE 9:**
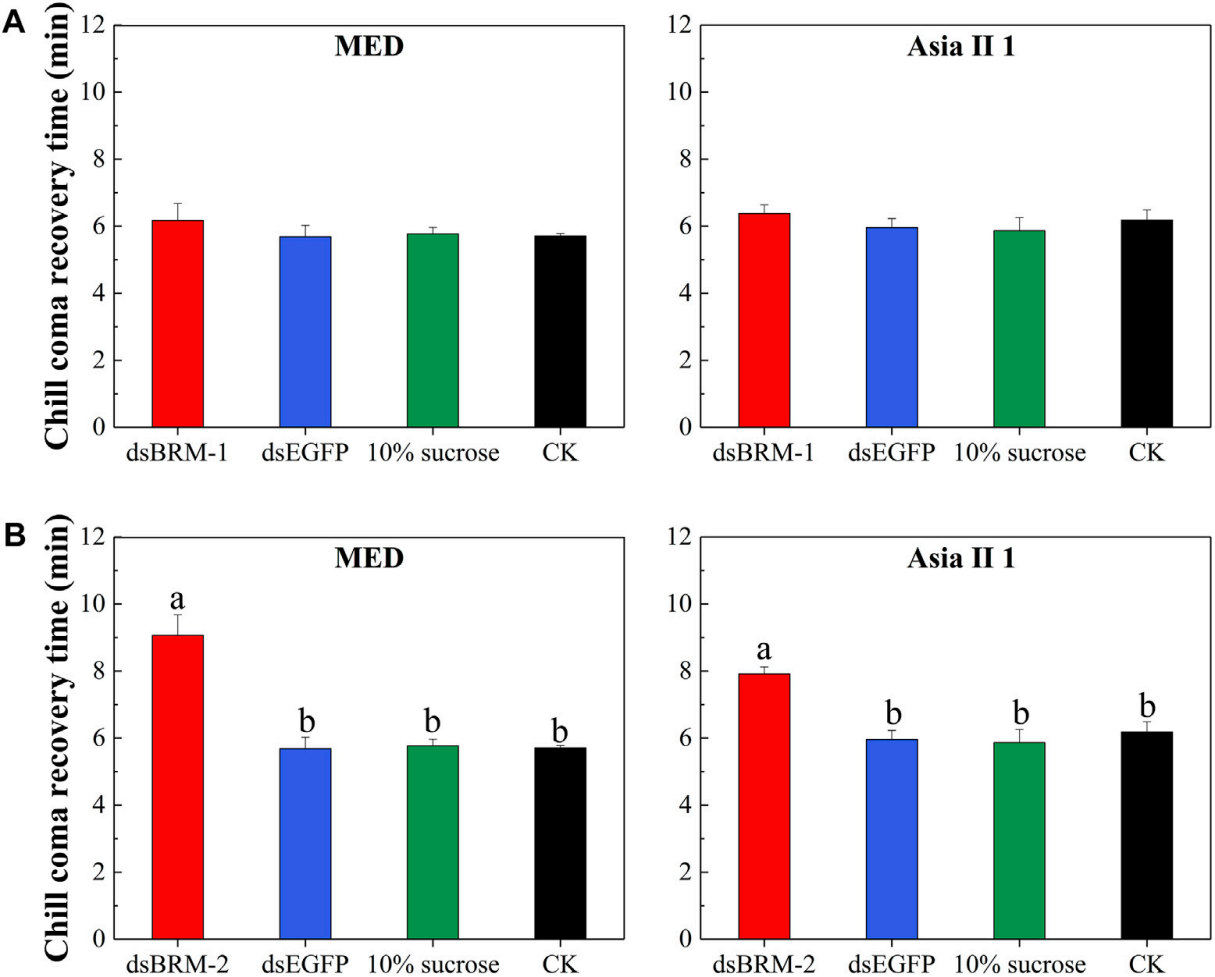
Mean chill coma recovery time (TRC) after double-stranded RNA (dsRNA) feeding in whiteflies. Compared with the controls (whiteflies untreated (CK), given dsEGFP, or 10% sucrose), TRC for adults feeding with dsBRM-2 **(B)** was significantly increased. Although the TRC of MED and Asia Ⅱ 1 increased after silencing *Bt-BRM-1* **(A)**, there was no statistically significant difference. Moreover, cold resistance of the two cryptic species was reduced differently after interfering with the remodelers (details are listed in Supplementary Table S4). The data are presented as the mean ± SE; *n* = 200. Bars with different lowercase letters are significantly different at *p* < 0.05.

Compared with the control treatments, silencing *Bt-BRM-1* and *Bt-BRM-2* reduced the heat resistance of MED by 16.10 and 13.34%, respectively, while the reduction in heat resistance in Asia II 1 was 31.17 and 30.82%, respectively (Figure 8 and Supplementary Table S3). In addition, silencing *Bt-BRM-2* reduced the cold resistance of MED by 59.40%, and the reduction of cold resistance in Asia II 1 was 32.89%, respectively (Figure 9 and Supplementary Table S4). However, there were no significant changes in the cold tolerance of the two cryptic species after silencing *Bt-BRM-1*.

## Discussion

It is known that chromatin remodeling factors are related to biological responses to environmental stress, and they have been well characterized in several model organisms, such as *Saccharomyces cerevisiae*, *Drosophila*, *X. laevis*, and *Arabidopsis thaliana* (Bao and Shen 2007b; Yadon and Tsukiyama 2011). It is also known that the subunit numbers of SWI/SNF family remodelers vary in different organisms; for example, yeast has 11 to 15 subunits and *Drosophila* has 8. Analogously, the number of ISWI family remodeler subunits is two or three in yeast and four in *Drosophila* (Clapier and Cairns 2009). However, such knowledge is limited in non-model organisms (especially invasive insects). In this study, we identified, for the first time, 13 SWI/SNF family members and 10 ISWI family members in the whitefly genome, and we analyzed their exon-intron structures and conserved motifs using bioinformatics methods. Previous studies have found that catalytic subunits of chromatin remodeling factors share a conserved ATPase domain (Clapier and Cairns 2009). Similarly, motif analysis indicated that Bt-BRM-1 to Bt-BRM-3 (the catalytic subunit of SWI/SNF complexes) and Bt-ISWI-1 to Bt-ISWI-4 (the catalytic subunit of ISWI complexes) all have this special functional domain, which is required for remodeling and serves as a DNA-translocating motor to break histone-DNA contacts (Clapier and Cairns 2009).

To explore the function of chromatin remodeling factors in temperature adaptation of invasive and native *B. tabaci* species, we cloned two key catalytic subunits of SWI/SNF complexes. By analyzing the cloned catalytic subunit sequences from whiteflies, we found different amino residues between invasive MED and native Asia II 1; nevertheless, the sequences in MED and Middle East-Asia Minor 1 (MEAM1, another invasive species, with phenotypic plasticity similar to MED but higher than that of native species) were identical (predicted: XP_018897204.1). The distribution regions of invasive MED and MEAM1 in China are also approximately similar (Liu and Liu, 2012). These results indicate that nonsynonymous mutations in chromatin remodeling ATPase may be related to the plasticity and geographic distribution of invasive and native whiteflies. In addition, we found that the differential amino acid residues in Bt-BRM-2-D1194 E and L1408A between invasive and native *B. tabaci* were located in the SnAC and Bromo domains, respectively. These structures interact specifically with histones and play a crucial role in chromatin remodeling (Clapier and Cairns 2009; Sen et al., 2011). Jabba et al. (2014) demonstrated that minimal changes in the functional domain of the amino acid sequence were sufficient to generate a wide range of thermal sensitivities. Therefore, we hypothesize that the presence of these key mutation sites in remodelers may be one of the important factors influencing the temperature tolerance of invasive *B. tabaci* species, thus affecting their geographical distribution and dispersal.

Insects are poikilothermic ectotherms, and subtle changes in ambient temperature can cause a series of physiological changes and affect their life-history parameters, such as their survival rate (Couret et al., 2014; Musolin 2007). The rapid adaptation of invasive MED to various environmental conditions is likely the main reason for its successful colonization and rapid invasion (Cui et al., 2008; Yu et al., 2012; Munoz-Valencia et al., 2016). Studies have shown that the ability of organisms to cope with environmental stresses, such as heat or cold, relies on rapid and suitable mechanisms for reprogramming gene expression (Asensi-Fabado et al., 2017). ATP-dependent chromatin remodelers (e.g., SWI/SNF complexes) have been found to affect chromatin structure and play an essential role in regulating gene expression (Bartholomew 2014). Previous studies have demonstrated that the expression of chromatin remodeling factors can be induced by temperature stress. For example, *CHR720* (SWI/SNF family) expression in *Oryza sativa* is clearly induced by cold stress (Hu et al., 2013). Similarly, in this study, the mRNA expression levels of catalytic subunits in whiteflies were found to be strongly altered under high or low temperatures. The expression patterns of *Bt-BRM-1* and *Bt-BRM-2* between MED and Asia II 1 were opposite under short-term heat stress. In addition, the mRNA levels of *Bt-BRM-1* and *Bt-BRM-2* in MED were significantly higher than those in Asia II 1 during heat treatment, and the expression level of *Bt-BRM-2* in MED was significantly higher than that in Asia II 1 during cold treatment. Thus, we speculate that the difference between the expression patterns of remodelers between invasive MED and native Asia II 1 might be related to the former’s phenotypic plasticity and adaptability in response to environmental stresses.

ATP-dependent chromatin remodeling factors play multiple roles in the temperature stress response. For instance, Erkina et al. (2010) reported that combinatorial inactivation of SWI/SNF and ISWI eliminated the preloading of HSF promoters, thereby affecting the synthesis of heat shock proteins. Research in *Drosophila* has shown that the chromatin-remodeling complex NURF cooperates with the GAGA factor to mobilize nucleosomes on the promoter of the heat-shock genes, thereby creating a nucleosome-free domain over the promoter, which exposes suitable sites for HSFs (Tsukiyama et al., 1995; Badenhorst et al., 2002). In addition, research in *Arabidopsis* has demonstrated that the chromatin-remodeling complex BRM contributes to transgenerational thermo-memory (Badenhorst et al., 2002). These experiments indicate that chromatin remodeling factors are involved in biological responses to temperature. In this study, we used the RNAi method to identify the functions of the remodelers under temperature stress conditions. The results showed that the two chromatin remodeling ATPase genes affected the heat tolerance of the two cryptic species, and *Bt-BRM-2* expression also affected the cold tolerance of the two cryptic species. Intriguingly, *Bt-BRM-2* had a greater impact on cold resistance in invasive MED, whereas *Bt-BRM-1* and *Bt-BRM-2* had a greater impact on heat resistance in native Asia II 1. Therefore, we speculate that *Bt-BRM-2* is an important regulatory factor for invasive MED diffusion to low temperature regions. However, the effects of the different expression patterns of the two chromatin remodeling elements on the specific regulatory mechanisms of temperature resistance between invasive MED and native Asia II 1 remains to be further studied.

To the best of our knowledge, this study is the first to identify and analyze the molecular characteristics of SWI/SNF and ISWI family members and reveal their possible key roles in temperature resistance in ectotherms. This study assists in understanding the adaptation mechanisms of invasive insects and enriches the body of literature focusing on stress from an epigenetic perspective.

## Data Availability

The original contributions presented in the study are included in the article/Supplementary Material, further inquiries can be directed to the corresponding author.
