# Supplementary material for: Characterization of Chromatin Remodeling Genes Involved in Thermal Tolerance of Biologically Invasive Bemisia tabaci
Source: Front Physiol. 2022 May 20;13:865172. doi: 10.3389/fphys.2022.865172 (PMC9163341; doi:10.3389/fphys.2022.865172)
Supplement: Supplementary file 1 [file Table1.DOCX]

**Table S1**. Primers used for cDNA cloning, qPCR, and dsRNA synthesis.

| Primer | Primer Sequence (5’ to 3’) |
| --- | --- |
| Primers for full-length cDNA cloning |  |
| BRM-1-F1 | TTTGAAAGTGTTCTGCTC |
| BRM-1-R1 | TTCTAGAGTGGTGTCACG |
| BRM-1-F2 | AGTTGCCTGCACTCGACG |
| BRM-1-R2 | GAAGCAAACGATGCGATG |
| BRM-1-F3 | CGCATTACAATACATCGC |
| BRM-1-R3 | CAAGAACTGCTGCCGTTC |
| BRM-1-F4 | CACCCTGACAGCGAATAC |
| BRM-1-R4 | ATCGTGCCCCTGTAAGAC |
| BRM-2-F1 | TGGATAAACAGAAACACG |
| BRM-2-R1 | TTAAAGTCTTTACCGTGC |
| BRM-2-F2 | CAACTGGGATCGACCCTA |
| BRM-2-R2 | CCTTTGCGGAACAACATA |
| BRM-2-F3 | TGGGTGTTGGAGTTTGAA |
| BRM-2-R3 | GCCGTTTGCGAGTTTTCT |
| BRM-2-F4 | AAAGATGACGCAGAGGTT |
| BRM-2-R4 | ACGAGATACGAGTTGCTA |
| Primers for qPCR |  |
| BRM-1-QF | AACGCTTGGCTTTCCTCCTTTC |
| BRM-1-QR | ATCGCTTCCTCAGCATTCTCCC |
| BRM-2-QF | AAAACCATCCAAACAATCGC |
| BRM-2-QR | TTTCAAACTCCAACACCCAA |
| EF1-α-F | TAGCCTTGTGCCAATTTCCG |
| EF1-α-R | CCTTCAGCATTACCGTCC |
| β-tub-F | TGTCAGGAGTAACGACGTGTTTG |
| β-tub-R | TTCGGGAACGGTAAGTGCTC |
| Primers for dsRNA synthesis |  |
| BRM-1-DF | TAATACGACTCACTATAGGGCCTTGCTTATCGGACTC |
| BRM-1-DR | TAATACGACTCACTATAGGGATTGGGCTTGTTGTTTT |
| BRM-2-DF | TAATACGACTCACTATAGGGTGTGATATGTCAGGGCT |
| BRM-2-DR | TAATACGACTCACTATAGGGTACTCGGTGATTGGTGG |

**Table S2**. Details of BRM protein sequences used for phylogenetic analysis.

| Insect species | GenBank accession number | Insect species | GenBank accession number |
| --- | --- | --- | --- |
| *Sipha flava* | XP_025423084.1 | *Myzus persicae* | XP_022166801.1 |
| *Cyphomyrmex costatus* | XP_018396747.1 | *Vollenhovia emeryi* | XP_011868917.1 |
| *Trachymyrmex septentrionalis* | XP_018355410.1 | *Bombus terrestris* | XP_012166376.1 |
| *Nylanderia fulva* | XP_029162796.1 | *Camponotus floridanus* | XP_011257142.2 |
| *Leptinotarsa decemlineata* | XP_023029618.1 | *Apis mellifera* | XP_026300357.1 |
| *Anoplophora glabripennis* | XP_018562326.2 | *Apis dorsata* | XP_006622867.1 |
| *Photinus pyralis* | XP_031335174.1 | *Halyomorpha halys* | XP_014292008.1 |
| *Melanaphis sacchari* | XP_025204346.1 | *Polistes dominula* | XP_015191338.1 |
| *Tribolium castaneum* | EEZ97706.2 | *Rhopalosiphum maidis* | XP_026814829.1 |
| *Acyrthosiphon pisum* | XP_001947872.2 | *Agrilus planipennis* | XP_025830418.1 |
| *Sitophilus oryzae* | XP_030758143.1 | *Apis florea* | XP_012343937.1 |

**Table S3**. Heat resistance of MED and Asia II 1 was reduced differently after interfering with the remodelers.

**a**

| T_KD_ (min) | ds*BRM-1* | ds*EGFP* | 10% sucrose | CK | Resistance reduction (%) |
| --- | --- | --- | --- | --- | --- |
| MED | 26.16±0.80 | 31.18±1.30 | 31.24±1.22 | 31.34±1.05 | 16.10 |
| Asia II 1 | 16.73±0.27 | 24.50±0.60 | 25.53±1.62 | 25.13±0.90 | 31.17 |

**b**

| T_KD_ (min) | ds*BRM-2* | ds*EGFP* | 10% sucrose | CK | Resistance reduction (%) |
| --- | --- | --- | --- | --- | --- |
| MED | 27.02±1.04 | 31.18±1.30 | 31.24±1.22 | 31.34±1.05 | 13.34 |
| Asia II 1 | 16.95±0.71 | 24.50±0.60 | 25.53±1.62 | 25.13±0.90 | 30.82 |

**Table S4**. Cold resistance of MED and Asia II 1 was reduced differently after interfering with the remodelers.

**a**

| T_RC_ (min) | ds*BRM-1* | ds*EGFP* | 10% sucrose | CK | Resistance reduction (%) |
| --- | --- | --- | --- | --- | --- |
| MED | 6.17±0.51 | 5.69±0.34 | 5.77±0.20 | 5.71±0.07 | 8.44 |
| Asia II 1 | 6.38±0.26 | 5.96±0.27 | 5.86±0.40 | 6.18±0.31 | 7.05 |

**b**

| T_RC_ (min) | ds*BRM-2* | ds*EGFP* | 10% sucrose | CK | Resistance reduction (%) |
| --- | --- | --- | --- | --- | --- |
| MED | 9.07±0.61 | 5.69±0.34 | 5.77±0.20 | 5.71±0.07 | 59.40 |
| Asia II 1 | 7.92±0.21 | 5.96±0.27 | 5.86±0.40 | 6.18±0.31 | 32.89 |
